# FALLS IN ADULTS: STEADI AND DURABLE MEDICAL EQUIPMENT PREDICTIVE ANALYSIS

**DOI:** 10.1093/geroni/igae098.3366

**Published:** 2024-12-31

**Authors:** Karen Probst, Kathryn Unger

**Affiliations:** Gannon University, Erie, Pennsylvania, United States; Gannon University, Erie, Pennsylvania, United States

## Abstract

Falls are the leading cause of death with fall rates continuing to rise. The Center for Disease Control, (CDC, 2020) predicted that by the year 2030 there will be 53 million falls among older adults. This alarming rate of falls resulted in a comparative study of the Stopping Elderly Accidents, Deaths & Injuries (STEADI) assessments as well as the sensitivity and specificity of the tools. Research was conducted (n=116) to determine if a condensed 3-question questionnaire could provide a brief comprehensive evaluation of fall risk. The data was then analyzed to determine if the provision of specific durable medical equipment (DME) impacted the results of either STEADI assessment. The study involved distributing both versions of the STEADI electronically to participants at Chosen Mission Incorporated, a non-profit that distributes DME at no cost to their local community. The condensed STEADI was found to have a strong correlation to the comprehensive STEADI as well as the acquisition of DME. The assessment demonstrated a high sensitivity and low specificity indicating a connection between fall risk and actual falls within six months. The findings from this research may benefit healthcare professionals to better and more quickly identify individuals with increased fall risk and potential for falls within six months.

